# *LM*^2^*K* Model for Hosting an Application Based on Microservices in Multi-Cloud

**DOI:** 10.3390/s23094450

**Published:** 2023-05-02

**Authors:** Juliana Carvalho, Dario Vieira, Christiano Rodrigues, Fernando Trinta

**Affiliations:** 1 Information System Department, Federal University Piauí—(UFPI), Picos 64607-670, PI, Brazil; julianaoc@ufpi.edu.br; 2 Efrei Research Lab, EFREI Paris, 94800 Villejuif, France; 3 Computing Department, Federal University Ceará—(UFC), Fortaleza 60440-900, CE, Brazil; fernando.trinta@dc.ufc.br

**Keywords:** multi-cloud, microservice, cloud selection

## Abstract

Cloud computing has become a popular delivery model service, offering several advantages. However, there are still challenges that need to be addressed when applying the cloud model to specific scenarios. Two of such challenges involve deploying and executing applications across multiple providers, each comprising several services with similar functionalities and different capabilities. Therefore, dealing with application distributions across various providers can be a complex task for a software architect due to the differing characteristics of the application components. Some works have proposed solutions to address the challenges discussed here, but most of them focus on service providers. To facilitate the decision-making process of software architects, we previously presented PacificClouds, an architecture for managing the deployment and execution of applications based on microservices and distributed in a multi-cloud environment. Therefore, in this work, we focus on the challenges of selecting multiple clouds for PacificClouds and choosing providers that best meet the microservices and software architect requirements. We propose a selection model and three approaches to address various scenarios. We evaluate the performance of the approaches and conduct a comparative analysis of them. The results demonstrate their feasibility regarding performance.

## 1. Introduction

Cloud computing has emerged as a popular delivery model service in recent years. According to [1], it provides a large set of easily accessible and usable virtualized resources. We can dynamically reconfigure these resources to adapt to variable loads, enabling optimized resource utilization. Cloud providers typically use a pay-per-use model and must offer infrastructure guarantees through customized service-level agreements.

Applications built on cloud concepts offer benefits such as elastic growth, easy maintenance and updating, and efficient resource utilization through multi-tenancy approaches. However, building cloud-centric applications presents several challenges from a software engineering perspective. These obstacles encompass issues such as data privacy, migrating applications to the cloud, and vendor lock-in, a problem where applications are restricted to specific providers [2,3].

In recent years, several works have proposed solutions to the challenges related to developing native cloud applications, including those with system components distributed across multi-cloud providers. The distribution of these components considers requirements such as performance, availability, and operational costs of the service from the provider [4,5,6]. This scenario, known as a multi-cloud provider environment, provides software architects with various possibilities to set up applications while ensuring the service that best meets the application needs, regardless of which provider offers the service [7,8].

A multi-cloud provider environment encompasses different views on how applications use them. In this sense, different works proposed taxonomies and classifications, such as [8,9,10]. According to [11], there are three cloud service delivery models: multi-cloud, cloud federation, and inter-cloud. In this work, we adopted the multi-cloud service delivery model, as it does not depend on an agreement between providers and, consequently, offers a higher number of providers and cloud services.

Using multi-cloud providers can bring many economic benefits, especially if we consider the significant growth in the number of cloud providers and the number of resources they offer. However, many challenges remain, such as security, privacy, trust, legal issues, resource management, and the service-level agreement (SLA) of services [9]. In order to address these problems, we proposed PacificClouds [12], an approach that aims to manage the entire process of deploying and executing a microservice-based application distributed in a multi-cloud environment from the software architect’s perspective.

A microservice is an architectural style that has stood out in the design of cloud applications. According to [13], microservices decompose a monolithic business system into independently deployable services. Thus, we can build applications and organize codes in several different parts. Moreover, we execute them in separate processes, as described by [14]. The suitability of this architectural style for cloud applications also makes it a natural candidate for applications distributed in a multi-cloud environment. Therefore, in this paper, we focus on applications based on microservices.

In this paper, we propose a new provider selection model and three different approaches for this model. Unlike our previous works [15,16,17], we now consider that the communication time between microservices deployed in different providers must be handled. Next, we detail our main contributions.

1. We propose a new provider selection model named Link Microservice Mapped to the Knapsack ($LM^{2}K$).
2. We propose and implement three approaches for this model: (i) dynamic, (ii) greedy, and (iii) ant colony.
3. Unlike other works in the literature, our proposed model considers that a microservice can only be hosted by a single provider, decreasing the communication costs among cloud services.
4. We evaluate the performances of all approaches, compare the results obtained, and present each approach’s feasibility.
5. We expand the taxonomy provider and service granularity (PSG) [17]. It seeks to identify the number of providers and services before and after selecting providers for the models and approaches proposed in the literature.

The remainder of this paper is organized as follows. Section 2 presents the multi-cloud providers scenario and the vendor lock-in problem. Next, we present related works in Section 3. In Section 4 and Section 5, we present the $LM^{2}K$ model, the three proposed approaches, and their implementations. In Section 6, we describe the tools, scenarios, experiments, and discuss the results. Finally, in Section 7, we present our conclusions and future works.

## 2. Multiple Cloud Providers

Cloud computing proliferation occurs mainly due to its essential characteristics: resource pooling, broad Internet access, measured service, on-demand self-service, and rapid elasticity [18]. Moreover, cloud providers present the resources as if they are inexhaustible. In this model, users pay cloud providers based on resource usage and do not worry about infrastructure and service management, reducing operating costs for enterprises.

In this context, many cloud providers have emerged. In order to serve the largest number of customers and win them over, many providers have adopted specific strategies in how their users use resources. For this reason, users who want to make the most of cloud computing, either by using services from multiple providers to compose more complex and innovative applications, or by migrating from one provider to another to obtain better quality services, are required to rework their applications. In this way, the user becomes hostage to the cloud provider; this is also known as the [2] vendor lock-in problem.

One way to take advantage of cloud computing is to use multiple clouds. According to [11,19], a multi-cloud application uses geographically distinct cloud resources, sequentially or simultaneously. There are several terminologies for multi-cloud environments. Following our previous works, we adopt the definition presented in [11] and, therefore, we classify the delivery model used in this work as multi-cloud.

The resource management functions in multiple clouds are major challenges; they are responsible for (i) selecting resources in various clouds for the application deployment, (ii) monitoring the execution time of the application resources distributed across multiple clouds according to the functional and non-functional requirements, and (iii) monitoring the cloud providers’ resources. Resource management in multiple clouds can be thought of from the perspectives of software architects and providers. In this context, resource management plays an essential role in the distribution and execution of a distributed application and, consequently, in interoperability and portability. It makes the multi-cloud environment more flexible by offering the most appropriate resources to meet the requirements, both in the deployment and in the execution of an application.

## 3. Related Work

In this work, we propose the $LM^{2}K$ model to address the multi-cloud provider’s selection to deploy microservices from a software architect’s perspective. Furthermore, we propose three solutions: (i) a dynamic algorithm, (ii) a greedy algorithm, and (iii) an ant colony-based algorithm.

Other works in the literature also deal with the cloud provider selection problem. However, due to the number of open issues in this subject, the primary concern of these works is scattered across different aspects. This section describes and compares some research works that address the cloud provider selection problem from the software architect’s perspective and divides the works into three categories. The first category describes works that select services from a single cloud provider. The second presents works that select only one service among the services offered by multiple providers. Finally, in the third category, the works select various services from multiple providers.

### 3.1. Single Cloud for Service Selection

Reference [20] proposed a service composition using the eagle strategy with whale optimization algorithm (ESWOA). In this work, the authors used simple additive weighting (SAW) to classify candidate services, and user-defined requirement thresholds. The proposed solution only uses a sequential service composition structure but transforms the other types of structures into sequential structures. Ref. [6] presented the optimal fitness-aware cloud service composition (OFASC) using an adaptive genetic algorithm based on genotype evolution (AGEGA), which considers various quality of service (QoS) parameters. It provides solutions that satisfy the QoS parameters and the connectivity restrictions of the service composition.

Reference [21] proposed a structure for cloud service selections with criteria interactions (CSSCI) that applied a fuzzy measure and a Choquet integral to measure and aggregate non-linear relations between criteria. The authors used a non-linear constraint optimization model to estimate the interaction ratios of importance and Shapley’s criteria.

Reference [22] introduced a modified particle swarm optimization (PSO) approach based on QoS that reduces the search space for composing cloud services. They also proposed a modified PSO-based cloud service composition algorithm (MPSO-CSC), which removes the dominant cloud services and then employs the PSO to find the ideal cloud service set.

Reference [23] deals with the selection and dynamic composition of services for multiple tenants. The authors proposed a multi-tenant middleware for dynamic service composition in the Software-as-a-Service (SaaS) cloud. They presented a coding representation and adequacy functions to model service selection and composition as an evolutionary search.

### 3.2. Multiple Clouds for a Service Selection

The works described in this subsection used multi-cloud providers to select only one service from one cloud provider. Thus, despite using multi-cloud providers in the selection process, they did not select multiple services from multi-cloud providers or interrelate services and providers as in this work.

Reference [24] presented a classification-oriented prediction method that helps in the process of discovering candidate cloud services that generate greater user satisfaction. The approach proposed by the authors encompasses two primary functions: service classification by similarity and service classification prediction in the cloud, taking into account a user’s preference. For this, the authors used a cloud service classification prediction method called CSRP, which covers the identification of similar neighbors, customer preferences, customer satisfaction estimation, and classification prediction.

Reference [25] proposed a hybrid multi-criteria decision-making model for selecting services from the available providers. The methodology assigns several classifications for cloud services based on QoS parameters, using an extended gray technique for order preference by similarity to ideal solution (TOPSIS), integrated with the hierarchical analytical process (AHP).

### 3.3. Multiple Clouds for Multiple Service Selections

This subsection describes works that used multiple cloud providers in the selection process, and they selected multiple services from multiple providers. The works presented in this subsection selected only one service from each provider, while our models select microservices that require several cloud services.

Reference [26] proposed two task-scheduling algorithms in a cloud federation environment, both based on a common SLA. The algorithm receives a set of independent tasks and a set of m cloud providers, along with the runtime, gain, and cost penalty of tasks at different cloud providers. The problem is to schedule all tasks to the clouds concerning the SLA so that there is a balance between the overall processing time and the cost gain penalty.

Reference [4] proposed an automated approach for the selection and configuration of cloud providers for a microservice in a multi-cloud environment. The approach proposed by the authors uses domain-specific language to describe the requirements of an application’s multi-cloud environment and provides a systematic method for obtaining appropriate configurations that meet the requirements of an application and the restrictions of cloud providers. The solution proposed by [4] deals with cloud service selections composing microservices, in which the cloud services must be different cloud providers.

Reference [27] proposed a composite service selection (CSS) approach to address the configuration that involves multiple simultaneous requests for the composite service. The authors selected various service combinations in a multi-cloud environment. However, in our model, the services of the same combination can be in different cloud providers. Additionally, their work only deals with the sequential structure in each combination and does not consider the interactions among the selected combinations.

Reference [28] proposed a multi-objective hybrid evolutionary algorithm called ADE-NSGA-II to compose services in an inter-cloud environment, meeting users’ QoS requirements. In the proposed algorithm, the authors used adaptive mutation and a crossover operator to replace strategies of the genetic algorithm of non-dominated ordering-II (NSGA-II). The work of [28] differs from ours, as they dealt with the composition of services when selecting a service from each provider.

Table 1 summarizes the main characteristics of all related works presented in this section. The first two columns of the table enumerate and reference the related work. The third, fourth, and fifth columns use a taxonomy to identify the focus of the work concerning the number of providers and services used in each phase, which we call provider and service granularity (PSG). The third column shows the number of providers in the selection process, the fourth shows the number of selected providers, and the fifth indicates the granularity of services per selected provider. Column six presents the methods used to solve the selection problem, and the last column defines whether the user defines the requirements threshold. Furthermore, in Table 1, n indicates the number of providers in the selection process (n>1), and m indicates the number of providers selected by the selection process (1<m≤n).

## 4. Multi-Cloud Selection Model: ${\mathrm{LM}}^{2}K$

PacicClouds uses various functionalities to meet different goals, and one of its primary tasks is to select the available cloud providers that best serve application microservices. The main objective of this work is to choose cloud providers for hosting the application microservices. However, many cloud providers are available, and each provider offers several services. Furthermore, an application can have multiple microservices, each of which may require several cloud services to meet its needs. Therefore, selecting suitable cloud providers to host an application distributed in multiple clouds is a complex task.

This section proposes a new multi-cloud selection model for PacificClouds to host applications based on microservices, named Linked Microservice Mapped to Knapsack ($LM^{2}K$). This model focuses on applications by considering communications among microservices and mapping the multi-choice knapsack problem.

The proposed selection model selects cloud providers from the software architect’s perspective. Therefore, it is necessary to define their requirements before deploying an application. We assume that the provider’s capabilities are known. In this work, we focus on three requirements: (i) response time, (ii) availability, and (iii) application execution cost. However, our model can support other requirements, such as energy cost and different types of cloud resources (e.g., CPU), as long as the cloud provider provides that information, and the architect considers it an important feature.

First, we will describe the formalization of the service model. Then, we will explain the process of selecting providers. All the descriptions of equation nomenclature are summarized in Appendix A.

### 4.1. Formalization

The required cloud services used to compose a microservice have different requirements, and the providers offer many services with the same functionalities but with different capabilities. In this work, we chose to assess the application response time (execution time plus delay), cloud availability, and application execution cost, but other requirements can be included in our model. We use three user requirements that are sufficient to help one understand the proposed selection process. However, several other user requirements can be defined without significant changes in the code. In addition, the requirements for the communication links among the microservices must be added to all requirements.

**Definition** **1.** *Cloud service model —as S(S.rt,S.a,S.c), where S.rt is the response time, S.a is availability, and S.c is cost, as in [29].*

**Definition** **2.** *Cloud service class—as $SC_{l}$ = {$S_{l1},S_{l2}$, …, $S_{lo}$}, in which $S_{l1}$, $S_{l2}$, …, $S_{lo}$ are services of the same provider with the same functionalities but different capabilities.*

**Definition** **3.** *Service provider model—as $SP_{k}$ = {$SC_{k1},SC_{k2}$, …, $SC_{kp}$}, in which $SC_{k1}$, $SC_{k2}$, …, $SC_{kp}$ are service classes.*

**Definition** **4.** *Cloud provider set—as CP = {$SP_{1}$, $SP_{2}$, …, $SP_{q}$}, in which $SP_{1}$, $SP_{2}$, …, $SP_{q}$ are service providers.*

**Figure 1:**
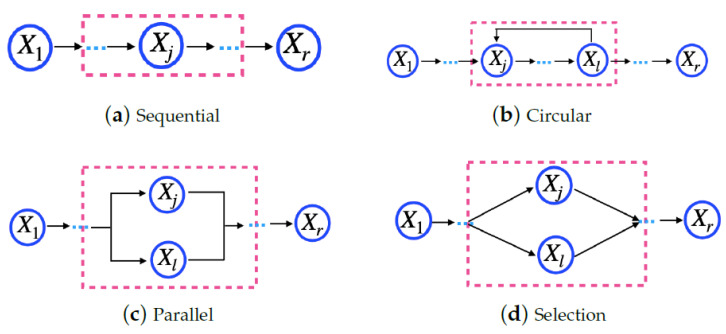
Structure of a microservice task.

**Definition** **5.** *Microservice model—as $MS_{i}$ = {$S_{i1}^{k_{1}},S_{i2}^{k_{2}}$, …, $S_{ir}^{k_{r}}$}, in which $S_{i1}^{k_{1}}$, $S_{i2}^{k_{2}}$, …, $S_{ir}^{k_{r}}$ are cloud services that are indispensable to executing a microservice, and they should be of different service classes. Thus, $1⩽k_{r}⩽o$, in which o is the maximum number of classes for a cloud provider. Moreover, a microservice consists of a task flow, and cloud services must perform it. Figure 1 shows four basic structures to compose a task: sequential, circular, parallel, and selection [30]. In Figure 1, $X_{v}$ indicates the $S_{iv}^{k_{v}}$, i.e., the service j from the provider k for the microservicei*.

**Figure 2:**
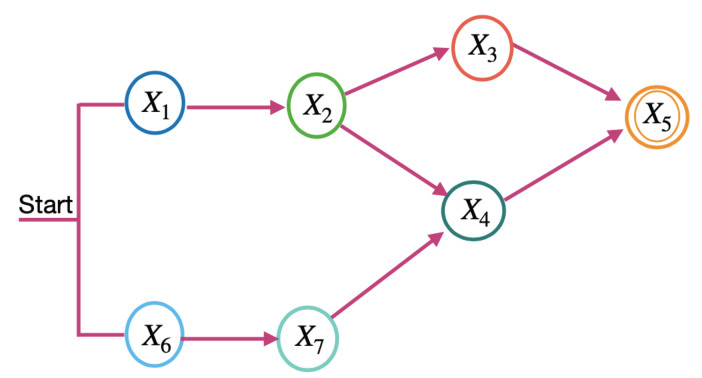
Task flow example.

*This work considers the task flow for a microservice, as shown in Figure 2. The task flow is an acyclic graph, in which each vertex represents a service, and each arrow represents a precedence relation between the services, following the basic structures shown in Figure 1. All cloud services used by a microservice must belong to the same provider. In Figure 2, $X_{v}$ indicates the $S_{iv}^{k_{v}}$, i.e., the service j from the provider k for microservice i* .

**Definition** **6.** *Application model—as AP = {$MS_{1},MS_{2}$, …, $MS_{t}$}, in which $MS_{1},MS_{2}$, …, $MS_{t}$ are microservices. For the application composition, microservices must use the basic structures shown in Figure 1. In Figure 1, $X_{v}$ indicates the $MS_{v}$, i.e., microservicev*.

*The application tasks are microservices. Therefore, an application task flow is a microservice flow. Figure 2 shows an example of a microservice flow considered in this work. In Figure 1, $X_{v}$ indicates the $MS_{v}$, i.e., microservice* *v*. *It differs from a single microservice task flow because the microservices of an application can be hosted on different cloud providers. In contrast, the cloud services required for a microservice must belong to the same provider*.

**Figure 3:**
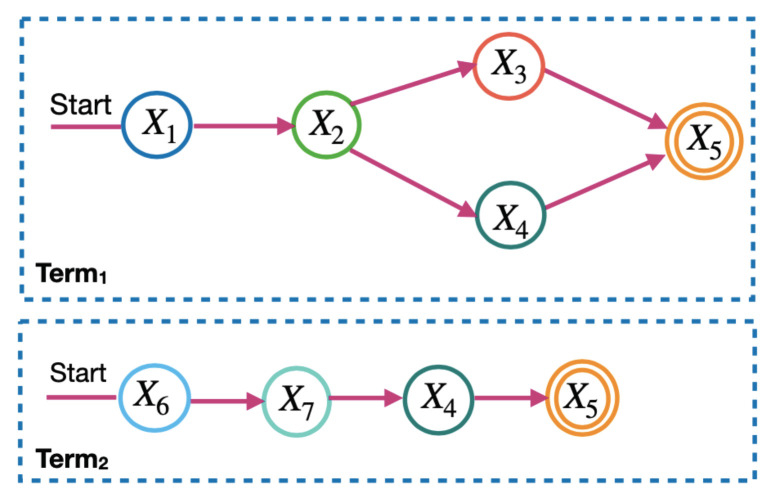
Terms of the microservice flow.

**Figure 4:**
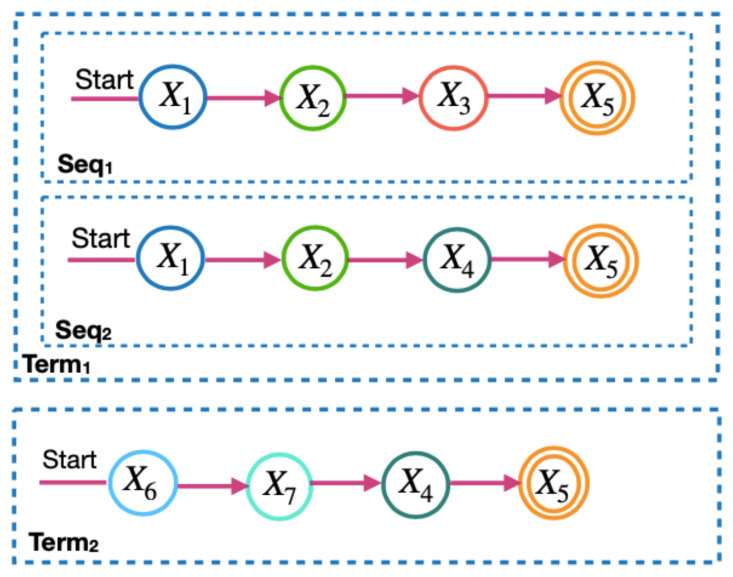
The sequences of the terms of the microservice flow.

*To calculate the microservice requirements in the $LM^{2}K$ model, the microservice flow is divided into two levels: the term and the sequence. Figure 3 and Figure 4 show the terms and sequences of the example of the microservice flow in Figure 2.*

*In Figure 3, terms 1 and 2 are the microservice flows of Figure 2, which must be performed in parallel. In Figure 4, the term sequences are the microservice flows for a term and only one of them is activated each time the microservice flow is executed.*

The next subsections describe how to calculate the values required for application execution distributed across multi-cloud providers. For this, we consider the application microservice flow and communication links. We define communication links as the communication among microservices. The formulas for calculating the requirement values are in accordance with [30,31,32,33,34].

#### 4.1.1. Availability Requirement

The cloud provider availability for an application must at least meet the threshold set up by a software architect. MinAvbty is the minimum availability threshold set by a software architect. In (1), we define AP.a as the availability, which is the product among all the application terms. Moreover, (2) defines $Term_{j}.a$ as the application term j availability. The availability of $Term_{j}.a$ is the sum of all sequence availabilities that belong to term j. In (3), we name $Seq_{ij}.a$ as the availability of the sequence i of the term j, which is the product of all microservice availabilities, the availability of all communication links, and the sequence selection probability represented by α (3).

(1) $$\begin{array}{c} AP.a=\prod_{j=1}^{num(Term)}Term_{j}.a⩾MinAvbty,\\ \forall Term_{j}|Term_{j}\in AP \end{array}$$

(2) $$Term_{j}.a=\sum_{i=1}^{num(Seq)}Seq_{ij}.a,\forall Seq_{ij}|Seq_{ij}\in Term_{j}$$

(3) $$\begin{array}{c} Seq_{ij}.a=\alpha \times \prod_{k=1}^{num(MS)}MS_{k}.a\times \prod_{k=1}^{num(MS-1)}Links_{k}.a,\\ \forall MS_{k}|MS_{k}\in Seq_{ij},Link_{k}.ainSeq_{ij} \end{array}$$

We calculate the availability of a microservice in a similar way to the application availability. The difference is that we based the application availability on the microservice flow. In contrast, we based the microservice availability on the cloud service availability. Cloud services are the services needed to compose a microservice.

#### 4.1.2. Response Time Requirement

An application response time must meet the threshold set by a software architect. A software architect defines MaxRT as the maximum response time threshold, which is the sum of the maximum execution time threshold (MaxExecTime) and the maximum delay threshold (MaxDelay), as shown in (4). In (5), we define AP.rt as the response time of an application, which receives the longest response time of all terms in an application and must be less than or equal to MaxRT. Moreover, (6) defines $Term_{j}.rt$ as the response time for the term j, which is assigned the sum of the response times for all strings that belong to the term j; (7) represents the response time for the sequence i of the term j; (7) represents the response time sum of all microservices that belong to the sequence, and the delays between each microservice multiplied by the sequence selection probability. We represent the sequence selection probability by α. We represent the delay between two microservices in a sequence of $Link_{k}.rt$.

(4) $$\begin{array}{c} MaxRT=MaxExecTime+MaxDelay \end{array}$$

(5) $$\begin{array}{c} AP.rt=\max_{j=1}^{num(Term)}\left\{Term_{j}.rt\right\}⩽MaxRT,\\ \forall Term_{j}|Term_{j}\in AP \end{array}$$

(6) $$Term_{j}.rt=\sum_{i=1}^{num(Seq)}Seq_{ij}.rt,\forall Seq_{ij}|Seq_{ij}\in Term_{j}$$

(7) $$\begin{array}{c} Seq_{ij}.rt=\alpha \times \left(\sum_{k=1}^{num(MS)}MS_{k}.rt+\sum_{k=1}^{num(MS-1)}Link_{k}.rt\right),\\ \forall MS_{k}|MS_{k}\in Seq_{jj},Link_{k}\in Seq_{ij} \end{array}$$

We calculate the response time for a microservice in a similar way to the application’s response time, but we based the application’s response time on the microservice flow. In contrast, we based the microservice response time on the cloud service response time. Cloud services are the services required to compose a microservice.

#### 4.1.3. Cost Requirements

The application execution cost should not exceed the budget, which is the cost threshold set up by a software architect. In (8), we define AP.c as the application execution cost, which is attributed to the total cost of all terms that belong to an application microservice flow and it must be less than or equal to the budget; (9) defines $Term_{j}.c$ as the term j cost, to which we assign the cost sum of all sequences belonging to the term j. We represent the sequence i cost from the term j in (10). Moreover, (10) receives the cost sum of all microservices that belong to the sequence and the communication links costs between each microservice multiplied by the sequence selection probability. We represent the sequence selection probability by α and the communication link cost between two microservices in a sequence by $Link_{k}.c$.

(8) $$\begin{array}{c} AP.c=\sum_{j=1}^{num(Term)}Term_{j}.c⩽Budget,\forall Term_{j}\\ |Term_{j}\in AP \end{array}$$

(9) $$Term_{j}.c=\sum_{i=1}^{num(Seq)}Seq_{ij}.c,\forall Seq_{ij}|Seq_{ij}\in Term_{j}$$

(10) $$\begin{array}{c} Seq_{ij}.c=\alpha \times \left(\sum_{k=1}^{num(MS)}MS_{k}.c+\sum_{k=1}^{num(MS-1)}Link_{k}.c\right),\\ \forall MS_{k}|MS_{k}\in Seq_{ij},Link_{k}\in Seq_{ij} \end{array}$$

We calculate the availability, response time, and cost for a microservice in a similar way to the application availability, response time, and cost, respectively. The difference is that we based the application availability, response time, and cost on the microservice flow. In contrast, we based the microservice availability, response time, and cost on the cloud service availability, response time, and cost, respectively. The cloud services are the services required to compose a microservice.

### 4.2. Cloud Provider Selection Process

This subsection details the cloud provider selection process for the model, which is divided into three levels. The first level discovers all candidate cloud services of a microservice across all cloud providers. The second level determines the candidate providers for all microservices of an application. The third level selects providers to deploy all microservices of an application. As in our previous work [15,16,17], this process is a combinatorial optimization problem that we mapped to the multiple-choice knapsack problem. Next, the three levels are described in detail.

#### 4.2.1. First Level

We select the services of each provider that meet all requirements of the software architects, which results in a set of candidate services for each provider. Next, we rank all candidate services in each provider. We use the simple additive weighting (SAW) technique as in [35], which has two phases: scaling and weighting.

- Scaling phase: First, a matrix $R=(R_{ij};1⩽i⩽n;1⩽j⩽3)$ is built by merging the requirement vectors of all candidate services. For this, the user requirements are numbered from 1 to 3, where 1 = availability, 2 = response time, and 3 = cost. The candidate services refer to the same microservice service. The entire process must be done for each microservice service. Each row $R_{i}$ corresponds to a cloud service $S_{ij}^{k_{j}}$ and each column $R_{j}$ corresponds to a requirement. Next, the requirements should be ranked using one of the two criteria described in Equations (11) and (12) in order to normalize the values. Equations (11) and (12) are used based on the requirements. For example, for the response time, the smaller, the better, contrary to availability, the greater, the better. Moreover, $R_{j}^{Max}=Max\left(R_{ij}\right),R_{j}^{Min}=Min\left(R_{ij}\right),1⩽i⩽n$.
  Negative: the higher the value ⇑, the lower the quality ⇓.
  (11) $$V_{ij}=\left\{\begin{array}{cc} \frac{R_{j}^{Max}-R_{ij}}{R_{j}^{Max}-R_{j}^{Min}} & \mathrm{if}\;R_{j}^{Max}-R_{j}^{Min}\neq 0\\ 1 & \mathrm{if}\;R_{j}^{Max}-R_{j}^{Min}=0 \end{array}\right.$$
  Positive: the higher the value ⇑, the higher the quality ⇑.
  (12) $$V_{ij}=\left\{\begin{array}{cc} \frac{R_{ij}-R_{j}^{Min}}{R_{j}^{Max}-R_{j}^{Min}} & \mathrm{if}\;R_{j}^{Max}-R_{j}^{Min}\neq 0\\ 1 & \mathrm{if}\;R_{j}^{Max}-R_{j}^{Min}=0 \end{array}\right.$$
- Weighting phase: All candidates will receive a weight, which is the overall requirements score, for each candidate cloud service (Equation (13)).
  (13) $$Score\left(S_{i}\right)=\sum_{j=1}^{3}\left(V_{ij}\times W_{j}\right)|W_{j}\in \left[0,1\right],\sum_{j=1}^{3}W_{j}=1$$

At the end of the first level, we must identify and classify all candidate services of a microservice for all available cloud providers.

#### 4.2.2. Second Level

We must build all candidate cloud service combinations from a provider to compose a microservice. For this, we must combine candidate services from a microservice that is offered by the same provider; (14) defines $SSP_{ik}$ as the set of candidate combinations from the provider k for the microservice i. In addition, in (14), $comb_{ikj}$ indicates the combination j from the provider k to the microservice i. In (15), a $comb_{ikj}$ combination is a set of cloud services from different classes to meet the microservice i needs. In (15), $S_{ij}^{k_{i}}$ indicates the candidate service j of the class j from the provider k needed for function j from microservice i. The microservice has a maximum of r functions and, consequently, requires a maximum of r cloud services, a candidate provider has a maximum of m combinations and a microservice has a maximum of q candidate providers.

(14) $$SSP_{ik}=\left\{comb_{ik1},\ldots ,comb_{ikm}\right\}$$

(15) $$comb_{ikj}=\left\{S_{i1}^{k_{1}},\ldots ,S_{ir}^{k_{r}}\right\}$$

We must calculate each combination score and cost in $SSP_{ik}$, as shown in (16) and (17). Moreover, (16) defines $comb_{ikj}.score$ as the combination of each service score sum plus the communication link score sum between each service that belongs to the service flow of the microservice. Furthermore, (17) defines $comb_{ikj}.c$ as the total cost of all services plus the cost of each communication link between services in a combination, according to the service flow of the microservice.

(16) $$comb_{ikj}.score=\sum_{x=1}^{r}Score\left(S_{ix}^{k_{x}}\right)+\sum_{x=1}^{r-1}Score\left(Link_{x}\right)$$

(17) $$comb_{ikj}.c=\sum_{x=1}^{r}S_{ix}^{k_{x}}.c+\sum_{x=1}^{r-1}Link_{x}.c$$

At the end of the second level, there is a set of all candidate combinations from all providers available for application microservices (SAMS), and each combination has a score and a cost. Moreover, (18) defines $SMS_{i}$ as a candidate providers’ set for the microservice i. In (18), $SSP_{ik}$ indicates the set of candidate combinations from the provider k to the microservice i. In (19), we define SAMS as the set of candidate providers for each microservice of the application. An application has a maximum of t microservices.

(18) $$SMS_{i}=\left\{SSP_{ik}|k\in \left[1,q\right]\right\}$$

(19) $$SAMS=\{SMS_{1},\ldots ,SMS_{t}\}$$

#### 4.2.3. Third Level

To host each microservice application, we must select the SAMS cloud providers (the result of the second level). For this, we must build candidate combinations for SAMS, so that each has a candidate combination for each microservice, and a set of these combinations is named SAPP (20). Moreover, in (20), $comb_{ik_{i}j_{k_{i}}}$ indicates the candidate combination j from the candidate provider k to the microservice i. Subsequently, (21) defines $SAPP_{l}.c$ as the execution cost for each combination in SAPP and checks whether it is less than or equal to the application execution cost, and excludes it otherwise. Next, we calculate each item score in SAPP. A candidate combination score ($SAPP_{l}.score$) is the score sum for each combination of items, as shown in (22).

(20) $$\begin{array}{c} SAPP=\{\left(comb_{1k_{1}j_{k_{1}}},\ldots ,comb_{tk_{t}j_{k_{t}}}\right)\\ |comb_{ik_{i}jk_{i}}\in SSP_{ik},SSP_{ik}\in SMS_{i},SMS_{i}\in SAMS,\\ 1⩽i⩽t,1⩽k_{i}⩽q_{i},1⩽j_{k_{i}}⩽w_{k_{i}}\} \end{array}$$

(21) $$SAPP_{l}.c=\sum_{i=1}^{t}comb_{ik_{i}j_{k_{i}}}.c+\sum_{i=1}^{t-1}Link_{k_{i}}.c$$

(22) $$\begin{array}{c} SAPP_{l}.score=\sum_{i=1}^{t}comb_{ik_{i}j_{k_{i}}}.score+\sum_{i=1}^{t-1}Link_{k_{i}}.score \end{array}$$

Summarily, the selection process first selects the services of all providers that meet all requirements, which results in a set of candidate services from all available cloud providers. Then, we assemble all combinations of candidate services from all providers that can be used to compose that microservice. Finally, we select one of the application combinations containing a provider to host each microservice based on a score. Therefore, as in our previous works [15,16,17], the selection process described for the $LM^{2}K$ model can be considered a combinatorial optimization problem. Hence, we mapped the selection process to the multiple-choice knapsack problem as in our previous works [15,16,17].

## 5. Approaches for the ${\mathrm{LM}}^{2}K$ Model

In this section, we present and describe our proposed approaches for the $LM^{2}K$ model. We mapped the selection process to the multi-choice knapsack problem and employed three algorithms suggested in the literature for this type of problem: dynamic (Section 5.1), greedy (Section 5.2), and ant colony (Section 5.3). We chose the dynamic algorithm to show an optimal solution, the greedy algorithm to accept larger data inputs, and the ant colony algorithm to obtain a better solution to the greedy (and a better data input than the dynamic). In this way, we can offer alternatives for the selection process according to the software architect’s needs.

### 5.1. Dynamic Approach

In this subsection, we present the example and the algorithm for the dynamic approach of the $LM^{2}K$ model.

#### 5.1.1. Dynamic Approach Example

**Figure 5:**
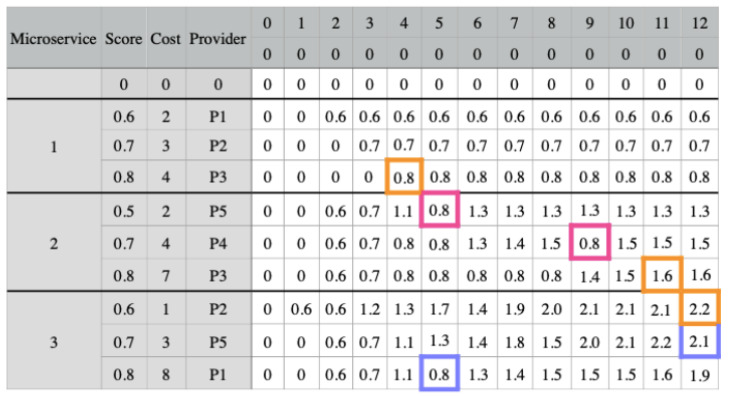
Dynamic approach example for $LM^{2}K$. The replaced scores are highlighted in pink. In blue are the scores that were affected by the altered scores. In orange is the selected combination for each microservice.

We present an example in Figure 5 that has three microservices and three candidate combinations for each one. The matrix presents the score, cost, and provider for each candidate combination. The software architect set a budget threshold of 12 for this application. Therefore, the matrix has 13 possible budgets (columns), ranging from 0 to 12. The filling of the matrix takes into account the communication links between the providers. We fill the last 13 columns by considering each candidate combination’s cost and score, as well as the potential application budgets.

In Figure 5, we fill the first column with zeros, as well as the first row, which is an extra row that does not include any combination. To fill in each cell for the first microservice combination, we check the combination’s cost. If it is higher than the budget value, we want to fill in the cell, we fill the cell with the previous cell value in the same column. Otherwise, we insert the combination score in the cell. The next step is to fill the cells corresponding to the other microservices. If the combination cost is higher than the budget value we want to fill, the cell value will be the highest value among the candidate combinations of the previous microservice from the same column.

In this example, we consider that the values of the requirements for providers P4 and P5 do not meet the application needs since we need to consider communication links. Thus, we notice that combination 1 for microservice 2 belongs to provider P5 and 2 to provider P4. In column 5 for combination 1, the value should be 1.2 (0.5 + 0.7), the combination score plus the highest score of microservice 1 in column 3 (5 − 2). Therefore, this cell receives the highest score from microservice 1, in column 5. The same applies to combination 2 for microservice 2 in column 9 (in pink). The values of these cells influence the combination values 3 and 2, from columns 5 and 12, respectively, for microservice 3 (in blue).

Finally, we must select a combination for each microservice. For this, we check the combination for the last microservice (microservice 3) and select the highest score, which is column 12 combination 1. Then, we update the budget by reducing the cost of microservice 3 from the budget (12 − 1). For microservice 2, we repeat the first step and search for the highest score on the new budget, which is combination 3 in column 11. Since the combination for microservice 2 is 7, we now have a budget of 4 (11 − 7). Lastly, for microservice 3, the highest score comes from combination 3, in column 4. The final result is highlighted in orange. Furthermore, we notice the influence of the communication link between the providers.

#### 5.1.2. Dynamic Algorithm

In this subsection, we describe the dynamic algorithm, divided into two parts as follows: the first part, illustrated by Algorithm 1, represents the first and second levels for the dynamic, greedy, and ant colony approaches; and the second part, which describes the third level and is illustrated by Algorithm 2.
**Algorithm 1:** Base Algorithm—Part 1.  **input**:   Set of the application requirements ap       Set of the available providers’ capabilities cp       Set of the communication links between services and providers lSer e lPr       Execution flow set between the microservice tasks and microservices fSer e fPr  **output**: Set of providers prvdsMs to host microservices

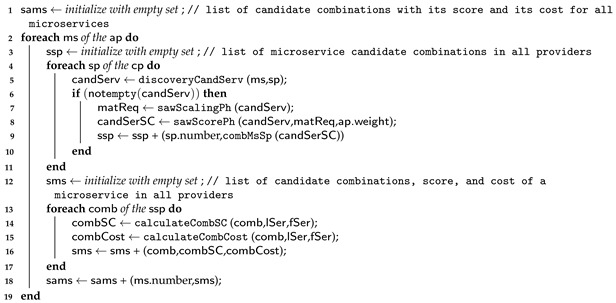



Algorithm 1 takes a five-set input: (i) a set of application requirements, (ii) a set of capabilities of the available providers, (iii) a set of communication links from the available providers, (iv) the execution flow set for the microservice functions, and (v) the set of application microservices. The algorithm searches for the available provider services that meet all microservice requirements on line 5. Then, it sorts them using SAW on lines 7 and 8. Next, on line 9, it combines the services so that each combination has all of the services that a microservice requires. This process is repeated for all available providers. Furthermore, Algorithm 1 calculates the score and cost for each combination on lines 14 and 15, respectively. The algorithm calculates each combination’s score and cost values, considering the microservice task execution flow, availability, response time, and cost value of each communication link between the services required for a microservice in the same provider. This process is repeated for all application microservices between lines 2 and 19. In the end, Algorithm 1 outputs a set of candidate combinations for each microservice (SAMS), each with a score and a cost.
**Algorithm 2:** Dynamic algorithm—Part 2.
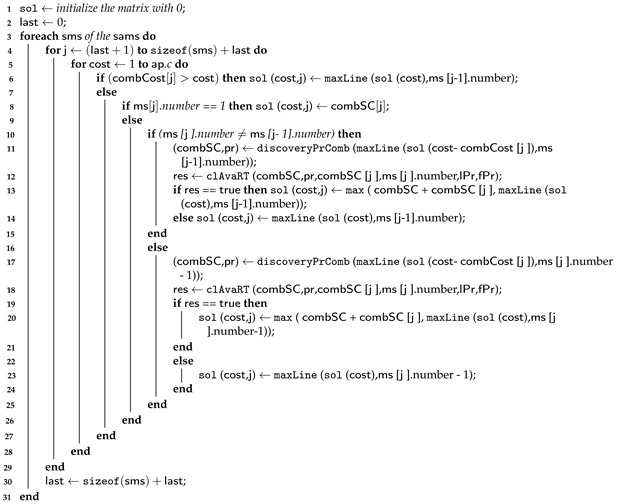


Algorithm 2 outputs a set of providers to host each application’s microservice. It represents the mapping of the selection process to the multi-choice knapsack problem. From lines 1 to 33, the algorithm selects a combination to host a microservice among the candidate combinations in SAMS (output of Algorithm 1). The algorithm creates a matrix (Sol) where each row contains a candidate combination for a microservice, and the columns represent the application budgets. The last column displays the budget threshold value defined by the software architect. The Sol Matrix is filled with the score values according to the candidate and the microservice it belongs to, ensuring that the combinations with the highest score, when combined, do not exceed the application’s budget (AP.c). Thus, Algorithm 2 selects a combination to host a microservice among the candidate combinations in SAMS between lines 1 and 33. This process is illustrated in the example in Section 5.1.1.

### 5.2. Greedy Approach

In this subsection, we describe the greedy approach to our proposed model. We present an example and the algorithm that contains the details of the selection process.

#### 5.2.1. Greedy Approach Example

**Figure 6:**
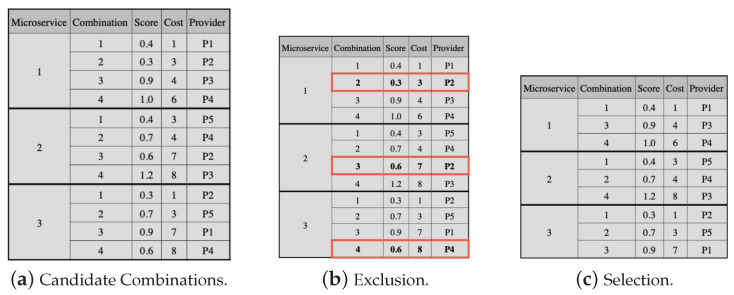
Greedy approach example for $LM^{2}K$—part 1. The combinations that should be excluded are highlighted in the figure.

Figure 6a presents the set of all candidate combinations for all application microservices, which has 4 candidate combinations for each of the 3 microservices.

Figure 6a shows the score, cost, and provider of each candidate combination. The combination cost is used to order candidate combinations for each microservice. It shows the exclusion of candidate combinations from each microservice that is dominated or lp-dominated described in [36]. In this example, combinations 2, 3, and 4 of microservices 1, 2, and 3, respectively (highlighted in the figure), have higher costs than the previous combination, even though they have a lower score. Therefore, these combinations should be excluded. Figure 6c shows the candidate combinations that are suitable for selection.

**Figure 7:**
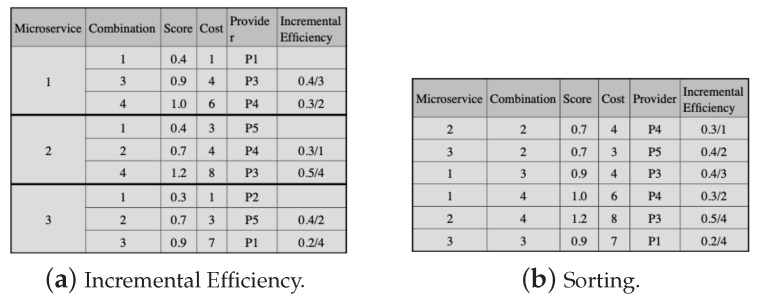
Greedy approach example for $LM^{2}K$—part 2.

The next step in the selection process is to calculate the incremental efficiency for all candidate combinations, except for the combinations with the lowest cost in each microservice, as shown in Figure 7a. Incremental efficiency is the cost–benefit of a candidate combination, and we use (23) to calculate it. In (23), i and j represent candidate combinations for a microservice, and j must be immediately after i. Moreover, sc and cost are the scores and the costs of the combinations. Then, the candidate combinations must be put in decreasing order, observing the incremental efficiency, except for the candidate combinations with lower costs for each microservice (in Figure 7b).

(23) $$e=\frac{sc_{j}-sc_{i}}{cost_{j}-costi}|1<j⩽num\left(comb\right),i⩽j$$

The lowest cost combinations for each microservice compose the initial solution, and we subtract their respective costs from the application budget. In the example, combination 1 of each microservice makes up the initial solution, and the application budget threshold is 12, with the lowest combination costs being 1, 3, and 1, respectively. We then check the possibility of replacing each solution combination with one from the set ordered by the incremental efficiency in Figure 7b.

**Figure 8:**
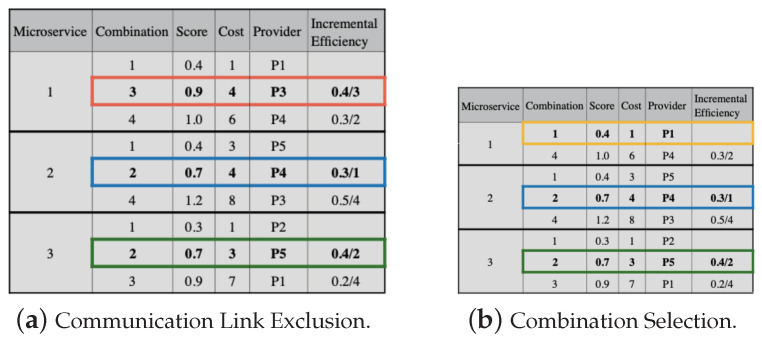
Greedy approach example for $LM^{2}K$—part 3. The selected combinations for Microservices 2 and 3 are highlighted in blue and green, respectively. In orange is the removed combination for Microservice 1, and in yellow is the selected combination.

To perform the replacement, we must observe the requirement values of the communication links between providers. Thus, in Figure 8a, we substitute combination 1 for 2, both for microservices 2 and 3. However, when combination 1 is replaced by 2 in microservice 1, the communication link values do not meet the application’s needs. Then, we must remove combination 2 for microservice 1. Next, we must continue the replacement process, but it is not possible, as the next value (line 5 in Figure 7b) exceeds the budget. Thus, combination 1 is selected for microservice 1, as shown in Figure 8b.

#### 5.2.2. Greedy Algorithm

Algorithm 1 represents the first and second levels for the three approaches of the $LM^{2}K$ model. Algorithm 3 illustrates the third level of the selection process using a greedy algorithm for the $LM^{2}K$ model.

In Algorithm 3, we classify candidate combinations for each microservice using the combination cost, line 2. From lines 4 to 8 and 9 to 13, we exclude each microservice’s dominated and lp-dominated candidate combinations according to [36]. Between lines 14 and 16, we subtract the first candidate combination cost from the budget for each microservice. From lines 17 to 27, we created an instance of the knapsack problem. For this, on line 20, we assign to SC the difference between the combination score j and the combination score j−1 for each microservice. On line 21, we assign to cost the difference between the combination cost j and the combination cost j−1 for each microservice. On line 22, we calculate the incremental efficiency; on line 23, we add to r a tuple containing the microservice identifier i, the candidate combination identifier j, the score resulting from line 20, the cost resulting from line 21, and the incremental efficiency e. Then, on line 28, we sort r in decreasing order according to the incremental efficiency.

After identifying the candidate combinations with the highest profit, we build the final solution by placing the selected combinations in Z. Thus, from lines 29 to 33, we add a tuple to *Z*, which contains the microservice identifier i, the provider identifier sms[0].sp, the combination identifier (in this case, it is equal to 1), and the score of the first candidate combination of a microservice. Then, we obtain the combinations to host each microservice, between lines 34 and 41. In line 36, we update the budget by subtracting the cost r[k] from it. Between lines 37 and 39, we compare the microservice identifier of r[k] with the microservice identifier of z. If the communication link requirements meet the application needs, we update the item score z with the item score r[k]. Finally, we obtain z, which contains all service combinations to host all microservices, and each combination can belong to a different provider. This process can be seen in the example of Section 5.2.1.
**Algorithm 3:** Greedy Algorithm—Part 2.
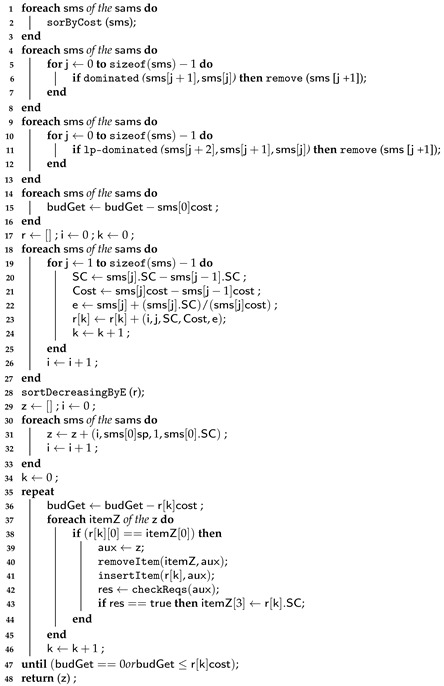


### 5.3. Ant Colony Approach

According to [37], the ant colony algorithm is a multi-agent system in which there is a low level of interactions among agents, known as ants, resulting in complex behavior similar to that of a real ant colony. The algorithm is based on the concept of modeling the problem as a search problem, where artificial ants search for the path with the lowest cost. Pheromone, a distributed information type, is modified by the ants to reflect their accumulated experience during problem-solving. The amount of pheromone in a given path influences the choices of the ants: the higher the pheromone level, the more likely an ant is to select that path. This indirect means of communication aims to provide information about the quality of the path components to attract other ants. In the following sections, we will present an example and provide details of the selection process.

#### 5.3.1. Ant Colony Approach Example

**Figure 9:**
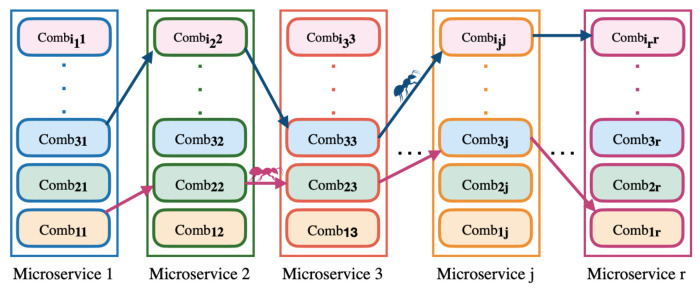
Example of the ant colony approach of model $LM^{2}K$.

For the $LM^{2}K$ model, each microservice represents a group of items, and each candidate combination ($Comb_{ij}$) is an item i of group j. Figure 9 shows the set of all candidate combinations for all application microservices, which has $i_{j}$ candidate combinations for each microservice j. For each candidate combination of Figure 9, we add the score, the cost, the provider to which it belongs, and the initial pheromone. We must order the candidate combinations for each microservice by the combination cost. The process has n ants, and each of them must build an individual solution. In each solution, we consider the response time, availability, and cost for the communication links between the providers of each candidate combination selected by an ant. We repeat the process (m cycles), and each ant builds a solution in each cycle, generating m solutions. The highest profit solution in each cycle is selected, and the best solution is selected by the end of all cycles.

#### 5.3.2. Ant Colony Algorithm

The algorithm for the ant colony approach has two parts, similar to the other approaches for the model. The first contains the first and second levels, and Algorithm 1 describes them. This subsection describes Algorithm 4, which represents the third level of the ant colony approach. The budget threshold is the knapsack capacity, and the pheromone is the profit.
**Algorithm 4:** Ant Colony Algorithm—Part 2.
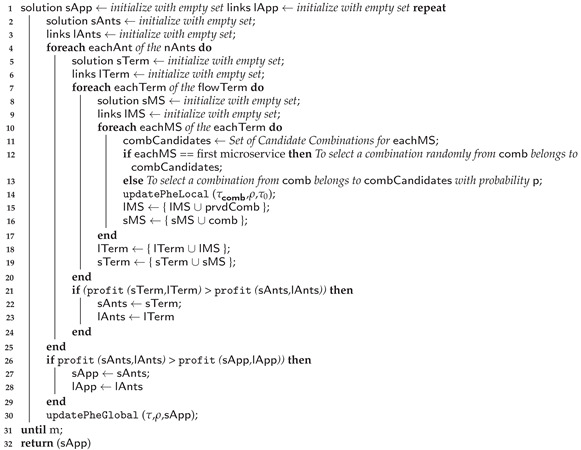


The input for this algorithm consists of the application execution flow set, a set of communication link capabilities between providers, and a set of all candidate combinations for each microservice obtained from the first and second levels. Furthermore, the algorithm initializes a set of pheromones for each combination, representing the initial pheromone level, denoted as $\tau_{0}$. In Equation (24), we assign $\tau_{0}$ as the sum of the product between the normalized requirement value and its respective priority. The normalized requirement value is obtained by dividing the requirement value by the range between the minimum and maximum values. Thus, AMin, RTMin, and CMin are the minimum values for each requirement (availability, response time, and cost), while AMax, RTMax, and CMax are the maximum values. Furthermore, priA, priRT, and priC are the priorities for the availability, response time, and cost, respectively. The process has m iterations and nAnts ants. In each cycle of this algorithm, the nAnts ants build individual solutions.

The microservices belonging to the application execution flow are the knapsack groups. Thus, the algorithm selects the first microservice candidate from the execution flow. The algorithm chooses the candidate combination from the first group randomly. Then, it updates the local pheromone, according to $\tau_{ji}$, as shown in (25), which is the local pheromone of candidate combination j from microservice i. In (25), we calculate $\tau_{ji}$ based on $\tau_{0}$ and the growth parameter called (ρ). The value of (ρ) must be between 0<ρ<1. For the other groups from the execution flow, the algorithm selects the most likely candidate combinations, according to (26).

An ant finishes building a solution when the solution contains a combination of each microservice from the execution flow. After each ant has created a solution, the algorithm identifies the best one for that iteration. Then, it updates the pheromone according to the best solution and according to τ, as shown in (27). We calculated τ based on ρ and the combination score. When the algorithm performs the maximum number of cycles, it ends.

The attractiveness of a combination depends on the pheromone deposited in it, and the combination selected in a group is the one that has the greatest attractiveness. The attractiveness of a combination is calculated by (26) [29]. In (26), β is a parameter that determines the relative importance of heuristic information and the pheromone level; the greater the β, the greater the influence of heuristic information in the selection process; $q_{0}$ is a parameter that determines the probability of choosing the best component and $0<q_{0}<1$; *q* is a random number in the range [0,1]; $\eta_{ji}$ is the score of the selected combination. According to [38], if $q_{0}=1$, ants will choose only the component with the highest value since $q_{0}$ will always be greater than *q*, which makes the algorithm a greedy search. However, if $q_{0}=0$, the algorithm will be more stochastic and less convergent.

(24) $$\tau_{0}=\frac{AMin}{AMax}\times priA+\frac{RTMin}{RTMax}\times priRT+\frac{CMin}{CMax}\times priC$$

(25) $$\tau_{ji}=\left(1-\rho \right)\times \tau_{ji}+\rho \times \tau_{0}$$

(26) $$p=\left\{\begin{array}{cc} max_{ji}\left\{\eta_{ji}^{\beta }\times \tau_{ji}\right\} & \mathrm{if}\;q⩽q_{0}\\ \frac{\eta_{ji}^{\beta }\times \tau_{ji}}{\sum_{ji}\left\{\eta_{ji}^{\beta }\times \tau_{ji}\right\}} & \mathrm{otherwise} \end{array}\right.$$

(27) τ=(1−ρ)×τ+ρ×score

## 6. Evaluation and Outcomes

In Section 4 and Section 5, we described the model and approaches for selecting multi-cloud providers to host a microservice-based application based on the software architect’s perspective. A single provider must host an application’s microservice that best meets the microservice requirements. To evaluate the performance of the proposed approaches for the $LM^{2}K$ model, in this section, we describe the tools implemented, the scenarios, and the experiments. Furthermore, we analyze the results obtained from the experiments.

### 6.1. Tool Description

We developed a tool for each proposed approach in the model. The tools were implemented using Python 3.7, and the input and output data were in the JavaScript Object Notation (JSON) format. For the $LM^{2}K$ model, the tool requires three input files. The first file is divided into two parts: one containing the requirements of the microservice-based application, and the other containing the capabilities of the available cloud providers. The second file includes the execution flow among the application’s microservices and the execution flow among the tasks within each microservice. The third file contains information about the capabilities of the interactions between the available cloud providers. The tool outputs a JSON file that contains the selected providers and services for hosting each microservice of the application.

### 6.2. Setting Scenarios

In this subsection, we describe the scenarios, experiments, and performance evaluation results for all approaches of the proposed model.

We conducted each experiment 30 times to ensure a normal distribution, as recommended by [39]. The scenarios were synthetically configured, aligning with common practices in the field, given the limited availability of datasets in the cloud computing research domain [40]. The providers in all scenarios were organized into sets based on the number of providers or provider services. We described twelve applications based on microservices, varying in the number of microservices, as presented in Table 2.

We assume that all microservices have the same size and require the same number of cloud services, but their requirements may vary. Each microservice requires three cloud services, which fall into different classes: computer, storage, and database. Consequently, the total number of cloud services required by an application is three times the number of microservices. Table 2 provides information on the number of microservices and the total services required for each configured application.

Most experiments use only five applications out of the twelve configured ones. Only experiments that check the influence of the number of microservices on the selection time of cloud services use most applications. Moreover, we set up three requirements for each microservice. The configured requirements are budget, response time, and availability, and we also determined the priority of each of them. We used them in all experiments and approaches. We performed the experiments on a MacBook Air with an i5 processor, 4 GB of RAM, and a 256 GB SSD.

The application budget is the maximum value available to hire all cloud services needed for an application. The sum of all cloud service costs from all cloud providers used by the application is the application cost. In this manner, we also defined the budget classes, each with a different application budget cost, but the service price remained the same for all applications. Furthermore, we determined the service prices based on the minimum and maximum costs of cloud services described for the providers’ capabilities.

We conducted experiments to explore variations in service costs by manipulating the budget classes. We defined availability and response time requirements within the capabilities of most providers. For instance, if we set the service cost to 10 and each microservice requires 3 services, the total cost for a single microservice would be 30. In a scenario where the application consists of 6 microservices, the budget would amount to 180. Similarly, for an application with 9 microservices, the budget would be 270. Both budgets fall into the same budget class. Alongside the budget class, we also established requirement classes with varying values. These classes were configured to decrease the response time and cost as the availability requirement increased. Thus, a higher requirement class value indicates a greater degree of restriction.

We present all experiment results in graphs, in which the y-axis shows the average provider selection time in milliseconds (s), the lines represent each application, and the x-axis shows the experiment.

### 6.3. Dynamic Approach Experiments

This section evaluates the performance of the dynamic approach of the $LM^{2}K$ model. To accomplish this, we established scenarios comprising the following components: (i) sets of providers (Table 3); (ii) capabilities of communication links among available providers; (iii) applications (Table 2); and (iv) the execution flow for each microservice application and its corresponding tasks, as described in Section 6.1. Table 3 presents eight sets of providers, with four sets differing in the number of providers and four sets differing in the number of services per provider. Furthermore, we configured the information pertaining to communication among the available providers, including availability, delay, and cost.

We evaluated the performance of the dynamic approach of the $LM^{2}K$ model through seven experiments. For this, we used 8 out of the 12 configured applications (Table 2) for the experiment that varied the number of microservices. The applications used in this experiment were APP1, APP3, APP5, APP7, APP9, APP10, APP11, and APP12. We also utilized five configured applications (APP2, APP3, APP4, APP6, and APP8) for other experiments. The availability, response time, and cost variation requirements depend on the type of assessment.

For each application, the software architect must define the budget threshold, availability, and response time in addition to their weight. Moreover, the architect must configure execution flows among the application’s microservices and each microservice. Thus, it must define each sequence among microservices and determine which microservices belong to the sequence, order, and frequency. A software architect must also specify the same information for the task sequences for each microservice.

**Figure 10:**
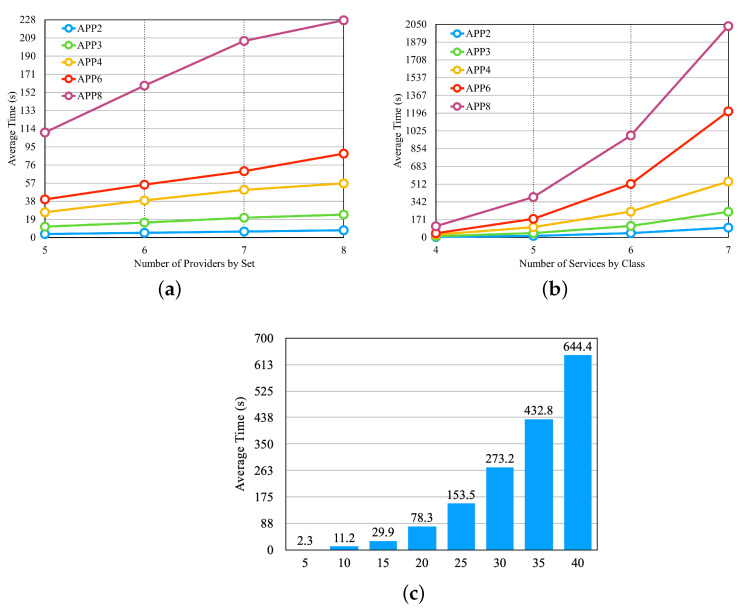
First three experiments of the dynamic approach—Table 3 sets. (**a**) Average Time per Set of Providers. (**b**) Average Time per Set of Provider Services. (**c**) Average Time per Microservices by Application.

From the configured scenarios, we defined experiments to evaluate the performance of the selection process. First, we conducted experiments by varying the number of providers, the number of services per provider, and the number of application microservices. In these experiments, the cost, availability, and response time requirements remained unchanged, and we defined values for them that should be met by most of the available providers. Figure 10 presents the results obtained from these experiments. In Figure 10a, we illustrate the results obtained by varying the number of providers. For this, we used the last four sets of providers from Table 3, which differ in the number of providers and are represented on the x-axis of Figure 10a. We can observe that the selection time increases significantly with an increase in the number of providers, but after a certain threshold, the growth rate decreases.

Figure 10b shows the result of the experiment, in which we vary the number of services per provider. In this experiment, we use the first four providers in Table 3, which differ in the number of services per provider and are presented on the x-axis of Figure 10b. We can notice that the increase in the average selection time is large when increasing the number of services by providers. Figure 10c illustrates the result of the experiment, in which we vary the number of application microservices, and we used the set of providers of the fifth line in Table 3. In Figure 10c, the x-axis shows the number of microservices by application. We can observe that the number of microservices influences the outcomes.

**Figure 11:**
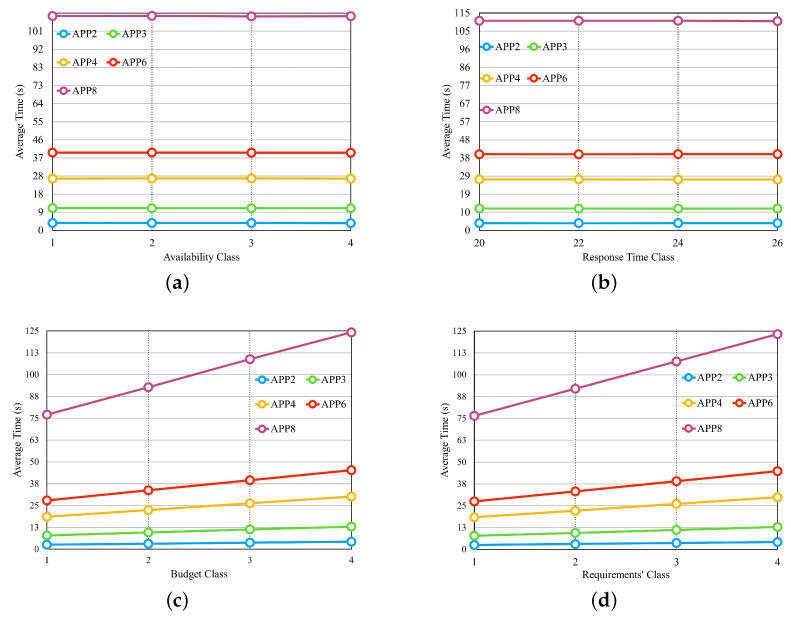
Four last experiments of the dynamic approach. (**a**) Average time for different classes of Availability. (**b**) Average time for different classes of Response Time. (**c**) Average time for different classes of the Budget Class. (**d**) Average time for different classes of the ’Requirements’ Class.

From the results obtained in the first three experiments, we selected the set of the fifth row from Table 3 for another four experiments, consisting of five providers, with each provider offering four services per class. Figure 11 illustrates the results of these four experiments in separate graphs. In the experiment shown in Figure 11a, we configure the availability requirement with four different classes, where each class has the same percentage of variation in individual values based on the number of microservices in each application. The cost and response time requirements are set to values that should be met by most of the available providers. We observed that the availability values did not significantly influence the average selection time. This result can be attributed to the fact that in order to meet the defined availability value, cloud services need to have high availability values. As a result, the number of services with such high availability does not vary significantly between different availability values. Figure 11b examines the performance of the approach when varying the response time requirement, with the applications configured to have four different values. The availability and cost requirements were set to values that should be met by most available providers. The results indicate that the response time values also do not significantly impact the average selection time, similar to the availability experiment.

In the experiment depicted in Figure 11c, we introduced budget classes as described in the preamble of Section 6.2. The results demonstrate that as the budget class increases, the average selection time also increases, which is in contrast to the findings of the previous two experiments. Therefore, the budget requirement has a significant impact on the average selection time. In the experiment shown in Figure 11d, where all three requirements are varied simultaneously, we created requirement classes as described earlier in Section 6.2. The results reveal that the average selection time increases as the requirements classes increase, primarily influenced by the cost requirement.

### 6.4. Greedy Approach Experiments

The purpose of this section is to verify the performance of the $LM^{2}K$ model’s greedy approach. To achieve this goal, we configured eight sets of providers, as shown in Table 4. Thus, four sets of providers differ by the number of providers, and four sets vary by the number of services per provider. In the sets that differ by the number of providers, each one has 25 services per class. The information defined for communication between providers includes delay, cost, and availability. We used 8 out of the 12 configured applications (Table 2) for the experiment that varied the number of microservices. The applications used in this experiment were APP1, APP3, APP5, APP7, APP9, APP10, APP11, and APP12. We used five of the configured applications for other experiments, i.e., APP2, APP3, APP4, APP6, and APP8. The requirements for availability, response time, and cost variation depend on the type of assessment.

**Figure 12:**
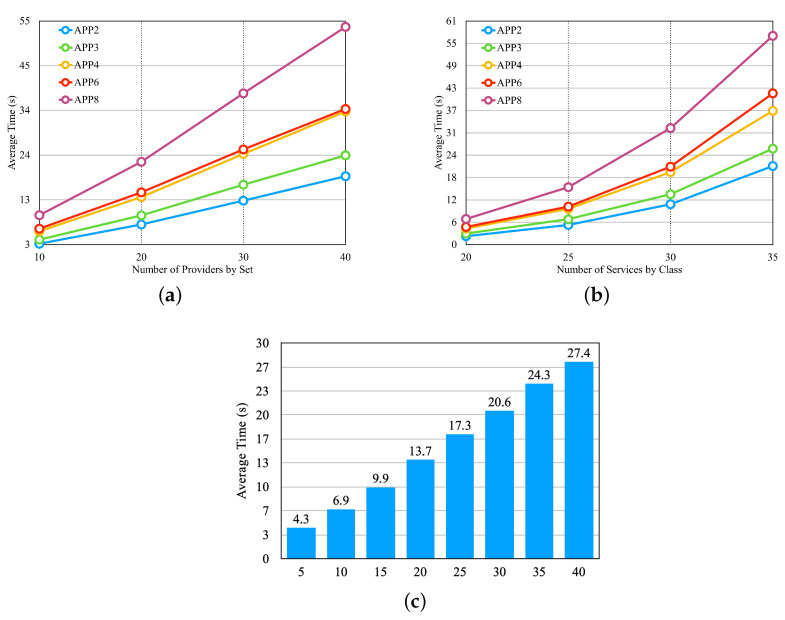
First three experiments for the greedy approach—Table 4 sets. (**a**) Average Time per Set of Providers. (**b**) Average Time per Set of Provider’s Services. (**c**) Average Time per Microservices by Application.

After setting up all of the scenarios, we performed three experiments (in Figure 12). The first experiment verified the performance of the approach by varying the number of providers, the second by varying the number of services by providers, and the third by varying the number of application microservices.

In the graphs, the x-axis shows the values related to the experiments. In Figure 12a, the x-axis shows the number of providers according to the first four lines of Table 4. In Figure 12b, the x-axis shows the number of services according to the last four lines of Table 4. In Figure 12c, the x-axis shows the number of microservices by application and the set of providers of the sixth line in Table 4. In the graphs in Figure 12, we notice that the average selection time increases with the increase in the number of providers, the number of services per provider, and the number of application microservices, respectively.

**Figure 13:**
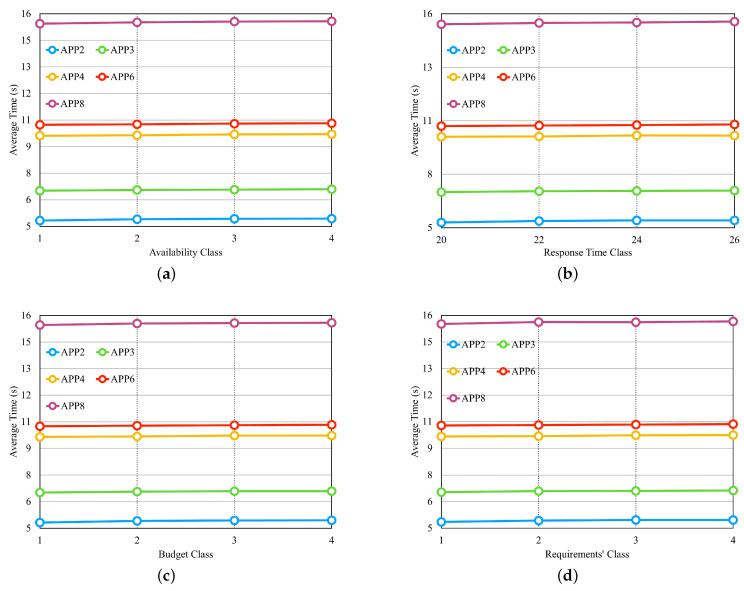
Four last experiments for the greedy approach. (**a**) Average time for different classes of Availability. (**b**) Average time for different classes of Response Time. (**c**) Average time for different classes of the Budget Class. (**d**) Average time for different classes of the ’Requirements’ Class.

From the first 3 experiments, we carried out 4 other experiments; we used a set of 15 providers, and each provider had 25 services per class, as described on the 5th line in Table 4. Figure 13 illustrates the experiments with a graph for each experiment.

In the experiment displayed in Figure 13a, there are four different classes for the availability requirement, in which each class has the same percentage, but the values vary individually according to the number of microservices in each application. We defined the response time and cost requirements with fixed values that can meet most providers. In the experiment in Figure 13b, there are four different values for the response time requirement. Availability and cost requirements remain fixed with values that can meet most providers. Figure 13c presents the result of the experiment, and it is possible to notice that none of the applications had an influence on the selection time with the variation of the budget classes, which were described in the preamble of Section 6.2. Figure 13d shows the result of the experiment, with three requirements modified at the same time so that when the availability requirement increased, the requirements of the response time and cost decreased. Thus, a smaller class is less restricted, and a larger one is more restricted. In Figure 13, we can observe that none of the experiments influenced the average selection time. The values differed only by application, as each one had a different number of microservices.

### 6.5. Ant Colony Approach Experiments

This section investigates the performance of the ant colony-bioinspired approach of the $LM^{2}K$ model. To achieve this, we define scenarios similar to the greedy approach of this model. Table 5 shows four sets of providers, which differ by the number of providers, and another four sets that vary in the number of services per provider. The communication between providers is characterized by delay, cost, and availability. We configured the applications following the same structure as the greedy approach of the proposed models, as presented in Table 2. Additionally, we define the execution flows to be the same as those in the greedy approach of the $LM^{2}K$ model, which were described in Section 6.4.

**Figure 14:**
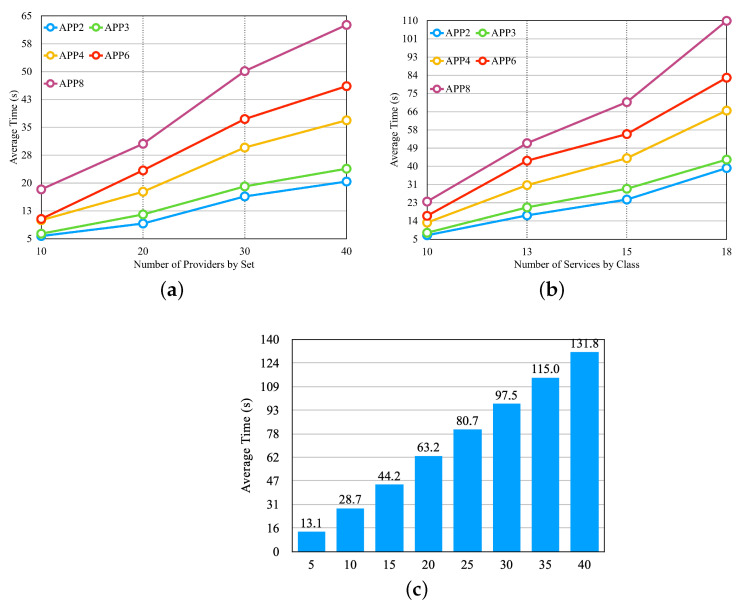
First three experiments for the ant colony approach—Table 5 sets. (**a**) Average Time per Set of Providers. (**b**) Average Time per Set of Provider’s Services. (**c**) Average Time per Microservices by Application.

After setting up the scenarios, we carried out three experiments to verify the performance of the approach by varying the number of providers, the number of services per provider, and the number of application microservices. Figure 14 presents the results of the three experiments, respectively.

In the graphs, the x-axis shows the values related to the experiments. In Figure 14a, the x-axis shows the number of providers according to the first four lines of Table 5. In Figure 14b, the x-axis shows the number of services according to the last four lines of Table 5. In Figure 14c, the x-axis shows the number of microservices by application and uses the set of providers from the sixth line in Table 5. In the graphs of Figure 12, we notice that the average selection time increases with the increase in the number of providers, the number of services per provider, and the number of application microservices, respectively. We also observe that the number of microservices per application has a greater influence on the increase in the average selection time.

**Figure 15:**
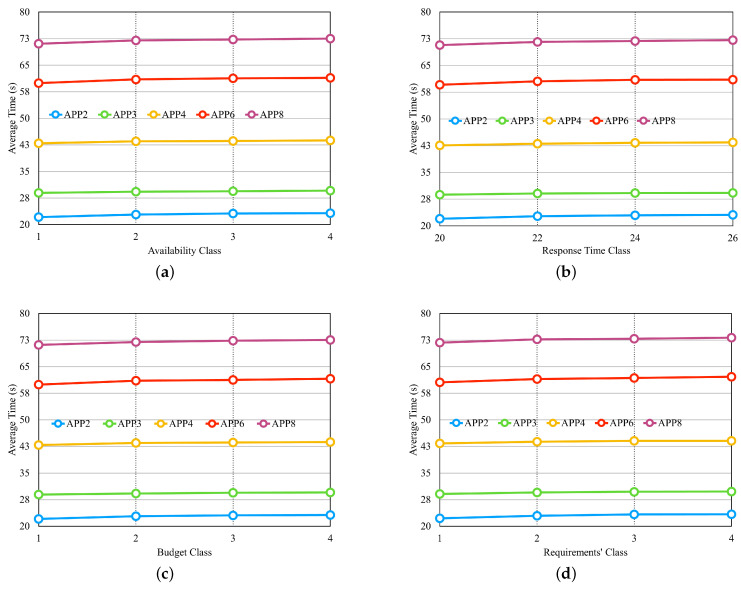
Four last experiments for the ant colony approach. (**a**) Average time for different classes of Availability. (**b**) Average time for different classes of Response Time. (**c**) Average time for different classes of the Budget Class. (**d**) Average time for different classes of the ‘Requirements’ Class.

We performed another 4 experiments, for which, we used a set with 15 providers, and each provider had 25 services per class as described in the fifth line in Table 5. Figure 15 shows a graph for each experiment. All experiments have the same configuration as the experiments performed for the greedy approach of the $LM^{2}K$ model presented in Section 6.4, except for the sets of providers. As in the experiments for the greedy approach, we notice that none of the experiments influenced the average selection time. The values differed only by application, as each of them has a different number of microservices.

### 6.6. Comparison of Outcomes

The $LM^{2}K$ model addresses applications based on microservices that depend on each other and require communication among them. Consequently, the calculation of application requirements distributed in multiple clouds needs to consider the microservice execution flow, including the availability, delay, and cost of communication between providers. Therefore, the approaches to the models need to address aspects that the other models did not consider. According to this context and the results obtained from the experiments, we observe that, in most outcomes, the average selection time does not change with the requirement variation. This result occurs because, in order to meet the thresholds defined by a software architect, providers must offer services with high values. Thus, the variation in the quantity of the offer has an insignificant amount in the average selection time. The exception is the dynamic approach for experiments that have budget variations, as they influence the average selection times and, thus, limit the size of the input for this approach. The greedy approach accepts a more considerable input than the ant colony approach, having an average time shorter than this. The ant colony approach allows for more significant input than the dynamic approach, and it also has shorter average selection times.

All experiments performed have varied at least one of the requirements or altered the number of providers per set or the number of services per provider. The experiments show the average time that each approach needs to select cloud providers to host the microservices of an application. We note that there is variation in the average selection time among applications because each application has a different number of microservices.

According to the experiments carried out, we also observed that there is a variation in the provider selection, the application requirement values, the number of providers, the number of services per provider, and even some changes in the provider’s capabilities, which allows us to obtain better offers. This result shows the sensitivity of the approaches proposed. Therefore, a software architect must adequately define the application requirement values to obtain better offers.

## 7. Final Consideration and Future Works

The growth of cloud computing and, consequently, the increased number of providers and services have facilitated the software development process. Moreover, these cloud providers have specialized their services in a way that increases the chance of a user becoming a prisoner to that provider, i.e., not being able to migrate to another provider or distribute an application across multi-cloud providers. For a software architect to maximize the benefits of cloud computing, it is necessary to encourage the use of multi-cloud providers, with each microservice hosted by the provider that best meets the application’s needs. Despite the many benefits of using multi-cloud providers, there are challenges, particularly in the absence of cooperation agreements among the involved providers in a multi-cloud environment.

One of the first decisions that a software architect must make for a traditional application is to choose the cloud providers to host each component, as the implementation depends on the provider selected. However, applications developed for the cloud must use an architectural style that brings greater flexibility, such as microservices. Thus, an application based on microservices facilitates its distribution to multi-cloud providers. Nevertheless, multi-cloud providers offer services with the same functionalities, but different capabilities, and each application’s microservice may need multiple cloud services. It is up to the software architect to perform the arduous task of selecting a service combination for each microservice from all available cloud providers.

In this work, we proposed a new model called Link Microservice Mapped to the Knapsack ($LM^{2}K$) and presented three approaches for selecting providers to host application microservices from the software architect’s perspective. These approaches include a dynamic algorithm, a greedy algorithm, and an ant colony algorithm.

The results showed that all proposed approaches are viable. However, each approach demands a different number of providers and application microservices. The ant colony’s algorithm cannot deal with input sizes as big as the greedy, though it usually provides better results than the greedy solution. Furthermore, the dynamic approach will provide the optimal solution but can only work with scenarios with a small number of providers, services, and microservices. Therefore, each approach has a particular suitability that depends on the number of providers, services, and microservices. Designing a specific scenario to compare them would not be a reasonable comparison.

In this work, we analyzed the approaches proposed for selecting multiple providers. Future work will involve conducting a more detailed analysis of the services and providers chosen in each approach. This analysis will examine how the selection may change based on variations in requirement values or priorities. Additionally, we plan to compare the proposed approaches based on the quality of the services selected in each experiment. The quality of services refers to the values and priorities of the requirements.

## Figures and Tables

**Table 1:** Summary of related work characteristics.

Items	Related Work	Characteristics				
		PSG			Method	User-Defined Threshold
		(1)	(2)	(3)		
1	[20]	1	1	Service Composition	ESWOA	Yes
2	[6]	1	1	Service Composition	OFASC and AGEGA	No
3	[21]	1	1	Service	Authors-defined	No
4	[22]	1	1	Service Composition	MPSO-CSC	Yes
5	[23]	1	1	Service Composition	Authors-defined	Yes
6	[24]	n	1	Service	CSRP	No
7	[25]	n	1	Service	Grey Technique, TOPSIS, and AHP	No
8	[26]	n	m	Tasks	Authors-defined	No
9	[4]	n	m	Service	Authors-defined	No
10	[27]	n	m	Service	Authors-defined	No
11	[28]	n	m	Service	Authors-defined	Yes
12	Dynamic $UM^{2}K$ [15]	n	m	Microservice	SAW, Dynamic Algorithm	Yes
13	Greedy $UM^{2}K$ [16]	n	m	Microservice	SAW, Greedy Algorithm	Yes
14	$UM^{2}Q$ [17]	n	m	Microservice	SAW, Authors-defined	Yes
15	Dynamic $LM^{2}K$	n	m	Microservice	SAW, Dynamic Algorithm	Yes
16	Greedy $LM^{2}K$	n	m	Microservice	SAW, Greedy Algorithm	Yes
17	Ant Colony $LM^{2}K$	n	m	Microservice	SAW, Ant Colony Optimization Algorithm	Yes

PSG—providers and services granularity, (1) provider granularity in the selection process, (2) provider granularity in the selection result, (3) service granularity per provider.

**Table 2:** Applications for all experiments.

Applications	Characteristics	
	Number of Microservices	Number of Services
APP1	5	15
APP2	6	18
APP3	10	30
APP4	14	42
APP5	15	45
APP6	18	54
APP7	20	60
APP8	22	66
APP9	25	75
APP10	30	90
APP11	35	105
APP12	40	120

**Table 3:** Set of providers for the dynamic approach.

Approach	Characteristics of Sets				
	Number of Sets	Number of Providers by Sets	Number of Services by Class	Number of Services by Provider	Number of Services by Set
Dynamic $LM^{2}K$	4	5	4	12	60
			5	15	75
			6	18	90
			7	21	105
	4	5	4	12	60
		6			72
		7			84
		8			96

**Table 4:** Set of providers for the greedy approach of $LM^{2}K$.

Approach	Characteristics of Sets				
	Number of Sets	Number of Providers by Sets	Number of Services by Class	Number of Services by Provider	Number of Services by Set
Greedy $LM^{2}K$	4	10	25	75	1500
		20			1500
		30			2250
		40			3000
	4	15	20	60	900
			25	75	1125
			30	90	1350
			35	105	1575

**Table 5:** Set of providers for the ant colony approach of $LM^{2}K$.

Approach	Characteristics of Sets				
	Number of Sets	Number of Providers by Sets	Number of Services by Class	Number of Services by Provider	Number of Services by Set
Ant Colony $LM^{2}K$	4	10	10	30	300
		20			600
		30			900
		40			1200
	4	15	10	30	450
			13	39	585
			15	45	675
			18	54	810

## Data Availability

Not applicable.

## Appendix A. Linked Microservice Mapped to the Knapsack Equation Nomenclature

**Table A1:** Description of equations’ nomenclature.

Description	Equation	Nomenclature
Availability (.a)	(1)	AP.a
Availability (.a) of *j*-th application term (.a)	(2)	$Term_{j}.a$
Availability (.a) of sequence *i* of the term *j*	(3)	$Seq_{ij}.a$
Maximum Response Time Threshold (.rt)	(4)	MaxRT
Response Time (.rt) of Application	(5)	AP.rt
Response Time (.rt) of *j*-th term	(6)	$Term_{j}.rt$
Response Time (.rt) of *i*-th sequence of the *j*-th term	(7)	$Seq_{ij}.rt$
Application Execution **Cost** (.c)	(8)	AP.c
Cost (.c) of *j*-th term	(9)	$Term_{j}.c$
Cost (.c) of *i*-th sequence of the *j*-th term	(10)	$Seq_{ij}.c$
Requirements Score of each candidate service	(13)	$Score(S_{i})$
Set of candidate combinations from the provider	(14)	$SSP_{ik}$
Combination (comb) of services for the *i*-th microservice	(15)	$comb_{ikj}$
Score (.score) of services’ combinations	(16)	$comb_{ikj}.score$
Cost (.cost) of services’ combinations	(17)	$comb_{ikj}.c$
Candidate providers’ set for the *i*-th microservice	(18)	$SMS_{i}$
Candidate combinations from *k*-th provider to *i*-th microservice	(18)	$SSP_{i}$
Set of all candidate combinations of providers for application microservices	(19)	SAMS
Candidate combination for each microservice	(20)	SAPP
Execution **Cost** (.c) for each SAPP combination	(21)	$SAPP_{l}.c$
Score (.score) for each SAPP combination	(22)	$SAPP_{l}.score$
Incremental **Efficiency** (*e*): cost–benefit of a candidate combination	(23)	*e*
